# Empathy Through Immersion: Exploring Staff Perceptions of Patient Experience Using Virtual Reality in Psychiatry

**DOI:** 10.1192/j.eurpsy.2026.10961

**Published:** 2026-07-31

**Authors:** G. Chaimowitz, D. Marginean, A. Olagunju, G. Behravan

**Affiliations:** St. Joseph’s Healthcare Hamilton, Hamilton, Canada

## Abstract

**Introduction:**

Virtual Reality (VR) technologies are emerging as powerful tools in healthcare education, as they offer immersive, controlled environments for experiential learning. Within forensic psychiatry, VR presents unique opportunities to cultivate empathy by allowing staff and trainees to experience scenarios from the perspective of patients, especially those in highly restrictive settings such as seclusion rooms and prison segregation.

**Objectives:**

This study examines healthcare professionals’ and students’ perceptions of two immersive VR scenarios, **Seclusion Suite** and **Prison Segregation**, designed to simulate the emotional and psychological challenges faced by individuals subjected to isolation as part of forensic psychiatric or correctional care.

**Methods:**

The study employs a descriptive qualitative design using semi-structured interviews with staff from St. Joseph’ Hospital, including nurses, physicians, and trainees. Each participant engages with one of the two VR scenarios before being interviewed. The **seclusion suite** scenario places users in the role of a patient undergoing involuntary seclusion, and highlights issues such as restricted autonomy, loss of dignity, and the critical importance of compassionate communication. The **prison segregation** scenario simulates the extreme isolation experienced by individuals in segregation, emphasizing the cumulative impact on mental health and the deprivation of basic human contact and environmental stimulation.

**Results:**

It is expected that findings will indicate these VR experiences significantly enhance participants’ empathic understanding and insight into the psychological toll of these interventions. It is hoped that participants express a deeper appreciation for patient vulnerability, the ethical complexities of isolation practices, and the emotional weight carried by patients in these environments. VR can provide a unique “first-person lens” that traditional training could not replicate. Themes emerging from the interviews may include increased sensitivity to trauma-informed care, awareness of power dynamics, and a renewed focus on dignity-preserving practices.

**Conclusions:**

By enabling perspective-taking and emotional engagement, VR training can enhance empathy, encourage reflection, and support ethically informed practice among healthcare professionals. The expected outcomes can have critical implications for staff training, policy development, and the broader goal of humanizing care in high-security and high-stress psychiatric environments. Future work will focus on scaling and refining these VR modules based on user feedback, as well as exploring measurable outcomes such as changes in staff behavior, communication style, and patient satisfaction.

**Disclosure of Interest:**

None Declared

